# Population-Based Surveillance of Birth Defects Potentially Related to Zika Virus Infection — 15 States and U.S. Territories, 2016

**DOI:** 10.15585/mmwr.mm6703a2

**Published:** 2018-01-26

**Authors:** Augustina Delaney, Cara Mai, Ashley Smoots, Janet Cragan, Sascha Ellington, Peter Langlois, Rebecca Breidenbach, Jane Fornoff, Julie Dunn, Mahsa Yazdy, Nancy Scotto-Rosato, Joseph Sweatlock, Deborah Fox, Jessica Palacios, Nina Forestieri, Vinita Leedom, Mary Smiley, Amy Nance, Heather Lake-Burger, Paul Romitti, Carrie Fall, Miguel Valencia Prado, Jerusha Barton, J. Michael Bryan, William Arias, Samara Viner Brown, Jonathan Kimura, Sylvia Mann, Brennan Martin, Lucia Orantes, Amber Taylor, John Nahabedian, Amanda Akosa, Ziwei Song, Stacey Martin, Roshan Ramlal, Carrie Shapiro-Mendoza, Jennifer Isenburg, Cynthia A. Moore, Suzanne Gilboa, Margaret A. Honein

**Affiliations:** ^1^Division of Congenital and Developmental Disorders, National Center on Birth Defects and Developmental Disabilities, CDC; ^2^Texas Department of State Health Services; ^3^Illinois Department of Public Health; ^4^Massachusetts Department of Public Health; ^5^New Jersey Department of Health; ^6^New York State Department of Health; ^7^North Carolina Department of Health and Human Services; ^8^South Carolina Department of Health and Environmental Control; ^9^Utah Department of Health; ^10^Florida Department of Health; ^11^University of Iowa; ^12^Puerto Rico Department of Health; ^13^Georgia Department of Public Health; ^14^Rhode Island Department of Health; ^15^Hawaii Department of Health; ^16^Vermont Department of Health; ^17^Division of Vector-Borne Diseases, National Center for Emerging and Zoonotic Infectious Diseases, CDC; ^18^Division of Reproductive Health, National Center for Chronic Disease Prevention and Health Promotion, CDC.

Zika virus infection during pregnancy can cause serious birth defects, including microcephaly and brain abnormalities (*1*). Population-based birth defects surveillance systems are critical to monitor all infants and fetuses with birth defects potentially related to Zika virus infection, regardless of known exposure or laboratory evidence of Zika virus infection during pregnancy. CDC analyzed data from 15 U.S. jurisdictions conducting population-based surveillance for birth defects potentially related to Zika virus infection.* Jurisdictions were stratified into the following three groups: those with 1) documented local transmission of Zika virus during 2016; 2) one or more cases of confirmed, symptomatic, travel-associated Zika virus disease reported to CDC per 100,000 residents; and 3) less than one case of confirmed, symptomatic, travel-associated Zika virus disease reported to CDC per 100,000 residents. A total of 2,962 infants and fetuses (3.0 per 1,000 live births; 95% confidence interval [CI] = 2.9–3.2) (*2*) met the case definition.^†^ In areas with local transmission there was a non-statistically significant increase in total birth defects potentially related to Zika virus infection from 2.8 cases per 1,000 live births in the first half of 2016 to 3.0 cases in the second half (p = 0.10). However, when neural tube defects and other early brain malformations (NTDs)^§^ were excluded, the prevalence of birth defects strongly linked to congenital Zika virus infection increased significantly, from 2.0 cases per 1,000 live births in the first half of 2016 to 2.4 cases in the second half, an increase of 29 more cases than expected (p = 0.009). These findings underscore the importance of surveillance for birth defects potentially related to Zika virus infection and the need for continued monitoring in areas at risk for Zika.

In 2016, as part of the emergency response to the Zika virus outbreak in the World Health Organization’s Region of the Americas, population-based birth defects surveillance systems monitored fetuses and infants with birth defects potentially related to Zika virus infection using a standard case definition and multiple data sources. Medical records were abstracted for data on birth defects, congenital infections, pregnancy outcome, head circumference, vital status, and Zika laboratory test results, irrespective of maternal Zika virus exposure or infection. Verbatim text describing the birth defects was reviewed to identify those that met the case definition. Infants and fetuses were aggregated into four mutually exclusive categories: those with 1) brain abnormalities or microcephaly; 2) NTDs; 3) eye abnormalities without mention of a brain abnormality included in the two previously defined categories; and 4) other consequences of central nervous system (CNS) dysfunction, specifically joint contractures and congenital sensorineural deafness without mention of brain or eye abnormalities included in another category. Because the evidence linking NTDs and congenital Zika virus infection is weak, prevalence estimates per 1,000 live births were calculated both overall and excluding NTDs for each quarter in 2016; CIs were calculated using Poisson regression (*1**,**2*).

All 15 U.S. jurisdictions^¶^ included in this report had existing birth defects surveillance systems that were rapidly adapted to monitor birth defects potentially related to Zika virus infection. These jurisdictions provided data on live births and pregnancy losses occurring from January 1–December 31, 2016. The jurisdictions were stratified into the following three groups: those with 1) confirmed local Zika virus transmission during 2016**; 2) one or more cases of confirmed, symptomatic, travel-associated Zika virus disease reported to CDC per 100,000 residents (i.e., “higher” Zika prevalence)^††^; and 3) less than one case per 100,000 residents of confirmed, symptomatic, travel-associated Zika virus disease reported to CDC (i.e., “lower” [low or no travel-associated] Zika prevalence)^§§^ (*3*).

Overall, 2,962 infants and fetuses with birth defects potentially related to Zika virus infection were identified (3.0 per 1,000 live births; CI = 2.9–3.2) (Table), including 1,457 (49%) with brain abnormalities or microcephaly, 581 (20%) with NTDs, 262 (9%) with eye abnormalities without mention of a brain abnormality, and 662 (22%) with other consequences of CNS dysfunction without mention of brain or eye abnormalities. Among the 2,962 infants and fetuses with defects potentially related to Zika virus infection, there were 2,716 (92%) live births. Laboratory evidence of possible Zika virus infection in maternal, placental, infant, or fetal specimens was present in 45 (1.5%) cases; 96 (3.2%) had negative tests for Zika virus, and 2,821 (95.2%) either had no testing performed or no results available.

**TABLE:** Population-based counts of cases of infants and fetuses with birth defects potentially related to Zika virus infection and prevalence per 1,000 live births — 15 U.S. jurisdictions,* 2016

Characteristic	Brain abnormalities or microcephaly^†^ (N = 1,457; 49%)	Neural tube defects and other early brain malformations^§^ (N = 581; 20%)	Eye abnormalities^¶^ (N = 262; 9%)	Consequences of CNS dysfunction** (N = 662; 22%)	Total (N = 2,962; 100%)
**Prevalence per 1,000 live births (95% CI)**	**1.5 (1.4–1.6)**	**0.60 (0.55–0.65)**	**0.27 (0.24-0.30)**	**0.68 (0.63–0.74)**	**3.0 (2.9–3.2)**
Eye abnormalities No. (%)	144 (9.9)	24 (4.1)	—	0	430 (14.5)
Consequences of CNS dysfunction No. (%)	133 (9.1)	77 (13.3)	12 (4.6)	—	884 (29.8)
**Pregnancy outcome^††^**					
Live births No. (%)	1,387 (95.2)	427 (73.5)	257 (98.1)	645 (97.4)	2,716 (91.7)
Neonatal death (≤28 days) No.	89	92	8	30	219
Pregnancy loss**^§§^** No. (%)	65 (4.5)	149 (25.6)	5 (1.9)	16 (2.4)	235 (7.9)
**Zika virus laboratory testing for infants or mothers**					
Positive No. (%)	29 (2.0)	4 (0.69)	10 (3.8)	2 (0.30)	45 (1.5)
Negative No. (%)	65 (4.5)	20 (3.4)	3 (1.1)	8 (1.2)	96 (3.2)
No testing performed/NA^¶¶^ No. (%)	1,363 (93.5)	557 (95.9)	249 (95.0)	652 (98.5)	2,821 (95.2)

**Abbreviations**: CI = confidence interval; CNS = central nervous system; NA = not available.

* 15 U.S. jurisdictions: Florida (selected southern counties), Georgia (selected metropolitan Atlanta counties), Hawaii, Iowa, Illinois, Massachusetts, New Jersey, New York (excluding New York City), North Carolina (selected regions), Puerto Rico, Rhode Island, South Carolina, Texas (Public Health Regions 1, 3, 9, and 11), Utah, and Vermont. Total live births = 971,685.

**^†^** Brain abnormalities or microcephaly (congenital microcephaly [head circumference <3rd percentile for gestational age and sex], intracranial calcifications, cerebral atrophy, abnormal cortical gyral patterns [e.g., polymicrogyria, lissencephaly, pachygyria, schizencephaly, gray matter heterotopia], corpus callosum abnormalities, cerebellar abnormalities, porencephaly, hydranencephaly, ventriculomegaly/hydrocephaly [excluding “mild” ventriculomegaly without other brain abnormalities], fetal brain disruption sequence [collapsed skull, overlapping sutures, prominent occipital bone, scalp rugae], other major brain abnormalities).

**^§^** Neural tube defects and other early brain malformations (anencephaly/acrania, encephalocele, spina bifida, and holoprosencephaly).

^¶^ Structural eye abnormalities (microphthalmia/anophthalmia, coloboma, cataract, intraocular calcifications, and chorioretinal anomalies [e.g., atrophy and scarring, gross pigmentary changes, excluding retinopathy of prematurity]); optic nerve atrophy, pallor, and other optic nerve abnormalities.

** Consequences of CNS dysfunction (arthrogryposis, club foot with associated brain abnormalities, congenital hip dysplasia with associated brain abnormalities, and congenital sensorineural hearing loss).

**^††^** 11 unknown pregnancy outcomes not included.

**^§§^** Includes miscarriages, fetal deaths, and terminations.

^¶¶^ Includes cases linked to lab data where no testing was performed or there was unknown testing status.

The prevalence of reported birth defects cases potentially related to Zika virus infection increased in jurisdictions with confirmed local transmission, from 2.8 per 1,000 live births (182 cases) during the first half of 2016 to 3.0 per 1,000 live births (211 cases) during the second half (CI = 2.4-3.2 and CI = 2.6–3.4, respectively; p = 0.10). In “higher” Zika prevalence jurisdictions, the monitored birth defects prevalence was 3.0 per 1,000 live births in both the first (753 cases) and second (775 cases) halves of 2016. In “lower” prevalence jurisdictions, the monitored birth defects prevalence declined significantly from 3.4 per 1,000 live births (549 cases) during the first half of 2016 to 3.0 (492 cases) per 1,000 live births during the second half (CI = 3.2–3.7 and CI = 2.8–3.3, respectively; p = 0.002) (Figure 1).

**FIGURE 1:**
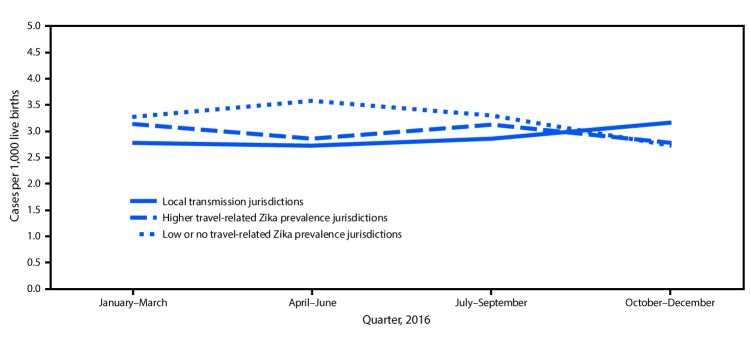
Prevalence of birth defects cases potentially related to Zika virus infection, by Zika virus transmission characteristics and quarter —15 U.S. jurisdictions, 2016*^,^^†,§^ * Local transmission jurisdictions included Florida (selected southern counties), Puerto Rico, and Texas (Public Health Region 11). **^†^** Higher travel-related Zika prevalence jurisdictions had one or more case of confirmed symptomatic travel-associated Zika virus disease reported to CDC per 100,000 residents. These jurisdictions included Georgia (selected metropolitan Atlanta counties), Massachusetts, New Jersey, New York (excluding New York City), Rhode Island, South Carolina, Texas (Public Health Regions 1, 3, and 9), and Vermont. **^§^** Low or no travel-related Zika prevalence jurisdictions had less than one case of confirmed symptomatic travel-associated Zika virus disease reported to CDC per 100,000 residents. These jurisdictions included Hawaii, Illinois, Iowa, North Carolina (selected regions), and Utah.

When NTDs were excluded, the prevalence of birth defects potentially related to Zika virus infection in jurisdictions with local Zika transmission increased 21%, from 2.0 per 1,000 live births (CI = 1.7–2.4) to 2.4 (CI = 2.1–2.8) (Figure 2). This increase indicated there were 29 more infants and fetuses with birth defects than were expected in areas with local transmission in the second half of 2016 (169 observed cases compared with 140 expected, p = 0.009). The prevalence of birth defects excluding NTDs in “higher” prevalence jurisdictions did not change (2.4 per 1,000 live births) and the prevalence in the “lower” prevalence jurisdictions significantly decreased from 2.8 per 1,000 live births (CI = 2.5–3.0) to 2.4 (CI = 2.2-2.7). Among 393 infants and fetuses with birth defects potentially related to Zika virus infection in areas with local transmission, 32 (8.1%) had laboratory evidence of possible Zika virus infection in a maternal, placental, infant, or fetal sample, 59 (15.0%) had negative Zika virus test results, and 302 (76.81%) had no testing performed or no results available.

**FIGURE 2:**
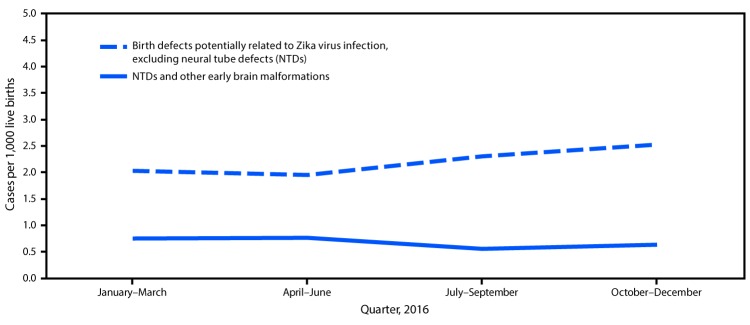
Prevalence of birth defects cases* potentially related to Zika virus infection in U.S. jurisdictions with documented local transmission of Zika virus,^†^ by defect type and quarter, 2016 *Fetuses and infants were aggregated into the following four mutually exclusive categories: those with 1) brain abnormalities with or without microcephaly (head circumference at delivery <3rd percentile for sex and gestational age); 2) NTDs and other early brain malformations; 3) eye abnormalities among those without mention of a brain abnormality included in the first two categories; and 4) other consequences of central nervous system dysfunction, specifically joint contractures and congenital sensorineural deafness, among those without mention of brain or eye abnormalities included in another category. ^†^ Jurisdictions with local transmission of Zika virus included Florida (selected southern counties), Puerto Rico, and Texas (Public Health Region 11).

## Discussion

Leveraging existing birth defects surveillance systems permitted rapid implementation of surveillance for birth defects potentially related to Zika virus infection early during the U.S. Zika virus outbreak. The prevalence of birth defects strongly linked to Zika virus infection increased significantly in areas with local Zika virus transmission (29 more than were expected in the second half of 2016 compared with observed prevalence in the first half). This finding underscores the importance of surveillance for birth defects potentially related to Zika virus infection and the need for continued monitoring in areas at risk for Zika transmission and exposure. 

An increase in birth defects potentially related to Zika was only observed in jurisdictions with local Zika virus transmission, and this difference was significant when NTDs were excluded. Brain and eye abnormalities and consequences of CNS dysfunction have been most consistently described in cases of congenital Zika infection, whereas the evidence supporting a possible association between NTDs and Zika virus infection during pregnancy is weak (*1*,*2*). In jurisdictions with “lower” (low or no travel-associated) Zika prevalence, the reason for the significant decrease in prevalence of birth defects potentially related to Zika (both including NTDs and excluding NTDs) is not clear. However, birth defects surveillance data typically are not final until approximately 24 months after the end of the birth year, and this release of data only 12 months after the end of the birth year likely resulted in less complete ascertainment of birth defects in late 2016 compared with early 2016. Further case ascertainment from the final quarter of 2016 is anticipated in all jurisdictions. In addition, the peak occurrence of birth defects potentially related to Zika virus infection is expected to have occurred in the 2017 birth cohort because the peak of Zika virus transmission occurred in Puerto Rico in August 2016, and local transmission of Zika virus was identified in southern Florida in June 2016 and in southern Texas in November 2016 (*4*–*7*).

The overall prevalence of the birth defects in this analysis (3.0 per 1,000 live births) was similar to a previously published baseline prevalence of birth defects potentially related to Zika virus infection from 2013–14 (2.9 per 1,000 live births; 95% CI = 2.7–3.1) (*8*). The findings presented here included data from an additional 12 jurisdictions, which covers a larger birth cohort totaling nearly 1 million live births, representing approximately one fourth of the total live births in the U.S. states and territories.

The findings in this report are subject to at least three limitations. First, the three jurisdictions with local Zika virus transmission differed from one another in the scope and timing of identified local transmission of Zika virus. Whereas Puerto Rico experienced a widespread outbreak that began in early 2016, local transmission in Texas was not confirmed until November 2016. In addition, jurisdictions with local transmission also had a high prevalence of travel-related Zika virus disease in 2016 (*3*), which could have contributed to the observed increased prevalence in birth defects. Second, increased awareness of birth defects potentially related to Zika virus infection in areas with local transmission might have resulted in increased efforts focused on rapid and complete identification of these birth defects cases during the second half of 2016. However, a significant increase in NTD prevalence was not observed. Although more complete ascertainment might partially explain the increased prevalence observed in areas with local transmission, it is unlikely that it would lead to a significant change, given the longstanding, mature surveillance systems, the standardized case review process, and no observable change in the prevalence of NTDs. Finally, jurisdictions in this analysis might differ in population demographics and systematic case-finding methodology, contributing to differences in observed prevalences among the three groups (*9*). A comparison of the prevalences in the first and second halves of the year was used to partially control for regional differences and monitor trends for those specific jurisdictional groups rather than to compare one group with another.

Collaboration between state and territorial Zika pregnancy and infant registries and birth defects surveillance systems provides a model for using the complementary approach of a prospective, exposure-based surveillance and conventional disease-based surveillance to respond to an emerging public health threat. The U.S. Zika Pregnancy and Infant Registry^¶¶^ can provide an early alert mechanism regarding clinical characteristics and manifestations of infants and fetuses with potential congenital infection; over 7,000 pregnancies with laboratory evidence of Zika virus infection have been reported, and CDC is monitoring pregnancy and infant adverse outcomes (https://www.cdc.gov/pregnancy/zika/data/pregnancy-outcomes.html). Established birth defects surveillance systems can adapt to monitor other emerging pregnancy, infant, and newborn outcomes of concern beyond structural birth defects, including functional problems such as hearing loss, and can provide additional clinical information through standardized data collection and clinical review. Finally, birth defects surveillance systems can provide an important mechanism for facilitating timely access to services among infants with birth defects and serve as a resource for assessing subsequent health and developmental outcomes among these children. The unique contributions of ongoing birth defects surveillance and the U.S. Zika Pregnancy and Infant Registry are both critical to optimally monitoring pregnant women and infants from the threat of Zika virus infection and implementing appropriate prevention efforts (*10*).

**Summary**

**What is already known about this topic?**

Data collected from three U.S. population-based birth defects surveillance systems from 2013 and 2014, before the introduction of Zika virus infection in the World Health Organization’s Region of the Americas, showed a baseline prevalence of birth defects potentially related to congenital Zika virus infection of 2.9 per 1,000 live births. Based on 2016 data from the U.S. Zika Pregnancy and Infant Registry, the risk for birth defects potentially related to Zika virus infection in pregnancies with laboratory evidence of possible Zika virus infection was approximately 20-fold higher than the baseline prevalence.

**What is added by this report?**

This report provides the first comprehensive data on the prevalence of birth defects (3.0 per 1,000 live births) potentially related to Zika virus infection in a birth cohort of nearly 1 million births in 2016. A significant increase in birth defects strongly related to Zika virus during the second half of 2016 compared with the first half was observed in jurisdictions with local Zika virus transmission. Only a small percentage of birth defects potentially related to Zika had laboratory evidence of Zika virus infection, and most were not tested for Zika virus.

**What are the implications for public health practice?**

Whereas the U.S. Zika Pregnancy and Infant Registry monitors women with laboratory evidence of possible Zika virus infection during pregnancy and their congenitally exposed infants, population-based birth defects surveillance systems make a unique contribution by identifying and monitoring all cases of these birth defects regardless of exposure or laboratory testing or results. Continued surveillance for birth defects potentially related to Zika virus infection is important because most pregnancies affected by Zika virus ended in 2017. These data will help communities plan for needed resources to care for affected patients and families and can serve as a foundation for linking and evaluating health and developmental outcomes of affected children.

## Footnotes

* With population-based surveillance for birth defects potentially related to Zika virus infection, information is collected on all infants who have birth defects that might be related to Zika virus infection. This includes infants who have not been exposed to Zika virus and might have the same birth defects for other reasons. This helps to identify the full spectrum of outcomes associated with Zika virus infection. https://www.cdc.gov/pregnancy/zika/research/birth-defects.html.

† Brain abnormalities or microcephaly (congenital microcephaly [head circumference <3rd percentile for gestational age and sex], intracranial calcifications, cerebral atrophy, abnormal cortical gyral patterns [e.g., polymicrogyria, lissencephaly, pachygyria, schizencephaly, and gray matter heterotopia], corpus callosum abnormalities, cerebellar abnormalities, porencephaly, hydranencephaly, ventriculomegaly/hydrocephaly [excluding “mild” ventriculomegaly without other brain abnormalities], fetal brain disruption sequence [collapsed skull, overlapping sutures, prominent occipital bone, and scalp rugae], and other major brain abnormalities); neural tube defects and other early brain malformations (anencephaly/acrania, encephalocele, spina bifida, and holoprosencephaly); structural eye abnormalities (microphthalmia/anophthalmia, coloboma, cataract, intraocular calcifications, and chorioretinal anomalies [e.g., atrophy and scarring, gross pigmentary changes, excluding retinopathy of prematurity]; optic nerve atrophy, pallor, and other optic nerve abnormalities); consequences of central nervous system dysfunction (arthrogryposis, club foot with associated brain abnormalities, congenital hip dysplasia with associated brain abnormalities, and congenital sensorineural hearing loss).

§ Neural tube defects and other early brain malformations are included as biologically plausible birth defects; however, they have been reported much less frequently with Zika virus infection than have defects in the other categories.

¶ Participating jurisdictions included Florida (selected southern counties), Georgia (selected metropolitan Atlanta counties), Hawaii, Illinois, Iowa, Massachusetts, New Jersey, New York (excluding New York City), North Carolina (selected regions), Puerto Rico, Rhode Island, South Carolina, Texas (Public Health Regions 1, 3, 9, and 11), Utah, and Vermont.

** Jurisdictions with confirmed local Zika virus transmission during 2016 were as follows: southern Florida, Puerto Rico, and Texas Public Health Region 11.

†† Jurisdictions with one or more cases of confirmed, symptomatic, travel-associated Zika virus disease reported to CDC per 100,000 residents (i.e., “higher” prevalence) included Georgia, Massachusetts, New Jersey, New York, Rhode Island, South Carolina, Texas Public Health Regions 1, 3, and 9, and Vermont.

§§ Jurisdictions with less than one case per 100,000 residents of confirmed, symptomatic, travel-associated Zika virus disease reported to CDC (i.e., “lower” prevalence) included Hawaii, Illinois, Iowa, North Carolina, and Utah.

¶¶ The U.S. Zika Pregnancy and Infant Registry includes the U.S. Zika Pregnancy Registry and the Zika Active Pregnancy Surveillance System, which together collect information about pregnancy and infant outcomes among women with laboratory evidence of Zika virus infection during pregnancy in the 50 states, the District of Columbia, and U.S. territories, until at least 2 years of age. (https://www.cdc.gov/pregnancy/zika/research/registry.html).
